# Effect of Exercise Repetitions on Arylesterase Activity of PON1 in Plasma of Average-Trained Men—The Dissociation between Activity and Concentration

**DOI:** 10.3390/antiox12061296

**Published:** 2023-06-17

**Authors:** Aneta Otocka-Kmiecik, Monika Orłowska-Majdak, Robert Stawski, Urszula Szkudlarek, Gianluca Padula, Szymon Gałczyński, Dariusz Nowak

**Affiliations:** 1 Department of Experimental Physiology, Interfaculty Chair of Experimental and Clinical Physiology, Medical University of Lodz, 6/8 Mazowiecka St., 92-215 Lodz, Poland; 2 Department of Clinical Physiology, Interfaculty Chair of Experimental and Clinical Physiology, Medical University of Lodz, 6/8 Mazowiecka St., 92-215 Lodz, Poland; 3 Academic Laboratory of Movement and Human Physical Performance, Medical University of Lodz, 251 Pomorska St., 92-213 Lodz, Poland

**Keywords:** PON1 activity, paraoxonase, arylesterase, PON1 concentration, physical activity, exercise, antioxidant

## Abstract

Exercise may increase the antioxidant capacity of plasma by stimulating antioxidant enzymes. The study aimed to measure the effect of three repetitions of acute exercise on arylesterase (ARE) activity of the paraoxonase 1 (PON1) enzyme. Eleven average-trained men (age 34.0 ± 5.2 years) completed three treadmill runs. ARE activity in plasma was evaluated spectrophotometrically and compared with PON1 concentration (PON1c), paraoxonase (PON) activity, and high-density lipoprotein cholesterol (HDL-C) at rest and after exercise. In all repetitions of the exercise, ARE activity remained stable, and ARE activity standardized for PON1c (ARE/PON1c) was lower post- than pre-exercise. The ARE/PON1c ratio changes returned to baseline levels during rest after each exercise session. Pre-exercise ARE activity correlated negatively with post-exercise C-reactive protein (CRP) (*ρ* = −0.35, *p* = 0.049), white blood cell count (WBC) (*ρ* = −0.35, *p* = 0.048), polymorphonuclear leukocytes (PMN) (*ρ* = −0.37, *p* = 0.037), and creatine kinase (CK) (*ρ* = −0.37, *p* = 0.036). ARE activity may be depleted under conditions of oxidative stress, as increases in PON1c during acute exercise did not result in parallel increases in ARE activity. No adaptation of the response of ARE activity to exercise was detected in subsequent exercise sessions. Individuals with lower pre-exercise ARE activity may develop a higher inflammatory response to strenuous exercise.

## 1. Introduction

The setting of exhaustive physical exercise offers an opportunity to observe changes that occur during acute oxidative stress. Exercise leads to a 10- to 20-fold increase in oxygen transport to the skeletal muscles [1,2]. In the milieu of a rise in metabolic rate, a decrease in plasma pH, and an increase in body temperature, the production of reactive oxygen species (ROS) accelerates, and the delicate redox balance may be disturbed. This may result in damage to cellular macromolecules, including DNA, lipids, and proteins. However, targeting endogenous modulators of antioxidant systems may restore the redox balance. Simioni et al. suggested that regular physical activity improves antioxidant defenses and lowers lipid peroxidation levels. [3]. It decreases the accumulation of oxidative proteins and DNA damage [4]. The surge of ROS produced during exercise may play an essential role in cellular homeostasis by activating a cascade of signaling molecules that enhance the gene expression of antioxidant enzymes [5]. Regularly repeated exercise may upregulate various antioxidant systems, including antioxidant enzymes such as superoxide dismutase, glutathione peroxidase, γ-glutamylcysteine synthetase (GCS) [6], and paraoxonase 1 (PON1) [7]. Furthermore, regular exercise repetitions have positively impacted the cardiovascular system by improving the plasma lipid profile, especially by increasing plasma high-density lipoprotein cholesterol (HDL-C) levels. HDL particles, primarily small, dense HDL3, show an atheroprotective effect by protecting low-density lipoprotein cholesterol (LDL-C) from free radical oxidative damage [8].

PON1 is an enzyme that may provide a link to the observed effect of improving plasma lipid profile and antioxidant status through regular physical activity. It is the only antioxidant enzyme that travels in the blood attached to HDL particles and possibly contributes to their anti-atherogenic properties. It hydrolyzes oxidized lipids in LDL, HDL, macrophages, and atherosclerotic lesions. It also exhibits lactonase activity toward homocysteine thiolactone (HCTL) [9]. The results of some studies indicate that not only HDL itself but also PON1 is an independent risk factor for coronary artery disease (CAD) [10,11]. PON1 activity was found to be much lower in patients after acute myocardial infarction than in healthy controls [12].

PON1 activity can be assessed by employing two substrates: paraoxon and phenylacetate. At first, the ability of PON1 to hydrolyze paraoxon and other organophosphates was described. In fact, the name paraoxonase (PON) comes from paraoxon, which is a toxic metabolite of the insecticide parathion. Therefore, initially, the enzyme became of great interest in toxicology. As PON1 was also found to hydrolyze aromatic esters, such as phenylacetate, the term arylesterase was introduced for this activity of the enzyme [7]. Both activities were found to have a role in the prevention of LDL oxidation and the accumulation of lipoperoxides in LDL [13]. While PON and ARE are independently related to cardiovascular risk in humans, ARE activity shows a higher prognostic value [14].

PON1 concentration and activity depend greatly on genetic factors. The gene is encoded on the long arm of chromosome 7 at q21-q22 in humans [15]. It has several polymorphisms. The most common ones include the one at position 192, which is a glutamine/arginine substitution (A→G) at position 192, and a methionine/leucine substitution (A→T) at position 55 [16]. The glutamine 192 (Q allele) and methionine 55 (M allele) isoforms were shown to offer greater protection against LDL oxidation than the arginine 192 (R allele) and leucine 55 (L allele) isoforms [13].

In addition to genetic variability, PON1 activity was found to be sensitive to different modulating factors, among which environmental, disease status, diet, and lifestyle interventions have been listed [17]. Some studies indicate that regular physical activity may regulate PON1 concentration and activity [18,19,20].

Our previous research shows that strenuous exercise results in a significant increase in PON activity and PON1 concentration in the plasma of young men, which contributes to the observed increase in the ferric-reducing activity of plasma (FRAP) [19,21,22,23]. This increase in PON activity is followed by a decrease, or at least a return to baseline levels, an hour and two hours after exercise. Similar results were obtained by Tomas et al. [24]. Studies on the effect of acute exercise on ARE activity are rather scarce and inconclusive, as a rise [19,21,22], no change [1,25,26], and a fall [27] in the activity after acute exercise was detected.

**Figure 1:**
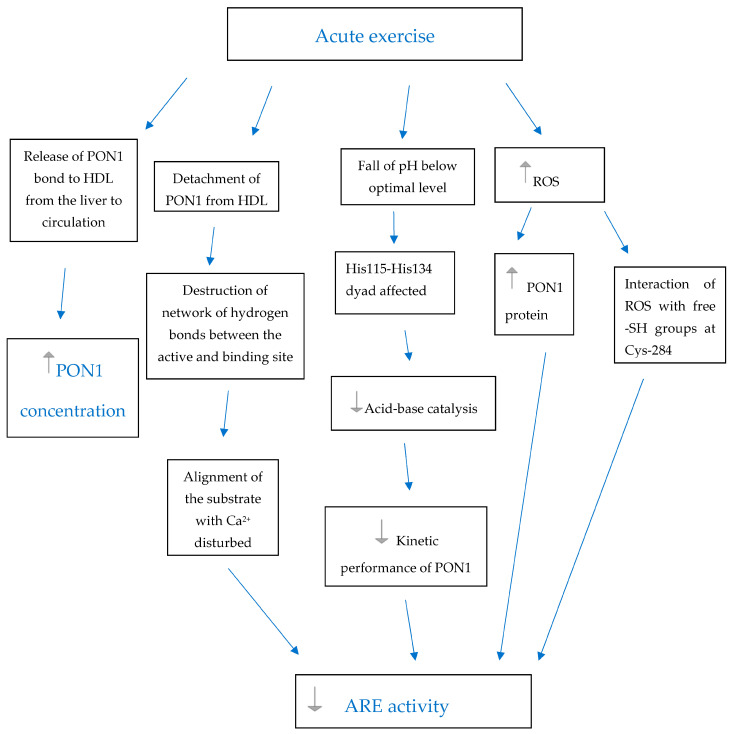
Plausible mechanisms of reversible increase in PON1 concentration with concomitant decrease in ARE activity in plasma during acute exercise. 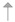-increase, 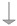
- decrease, PON1—paraoxonase 1, HDL—high-density lipoprotein, ROS—reactive oxygen species, Ca^2+^—catalytic calcium ion in the active site tunnel of PON1, His—Histidine, Cys—Cysteine, -SH groups—sulfhydryl groups.

In a previous study, we noticed that increments in PON activity were hampered by prolonged exercise [23]. The causative mechanisms of this activity inhibition may be associated with a fall in pH during strenuous exercise to a level that is below the optimal pH for PON1 activity. This would have a negative impact on the enzyme’s kinetic performance [28]. Acid–base mechanisms mediated by the His115-His138 dyad may be impaired, and substrate alignment with catalytic Ca^2+^ may be affected [29]. Furthermore, ROS may cause the oxidation of the PON1 protein. They may directly interact with free sulfhydryl (SH) groups in Cysteine 284, which is considered the active site of PON1 [30]. Exposition of the enzyme to oxygen-free radicals was shown to reduce the number of free SH groups, which was accompanied by a reduction in PON1 antioxidant activity. These putative mechanisms of PON1 inactivation are summarized in Figure 1.

The effect of regular training on PON1 activity is generally considered beneficial. In most studies, regular repetitions of exercise lead to an increase in ARE activity [1]. Sportsmen have higher ARE activity than sedentary subjects [31]. However, some studies failed to find the effect of regular physical activity on ARE activity [32]. Other results show that the influence of training on ARE activity varies depending on the PON1 polymorphism [33]. Training experience may also affect the response of ARE activity to exercise [19].

The mechanisms leading to changes in ARE activity in subjects who exercise regularly are not yet explained. To investigate the effect of training on ARE activity, we designed a study with three repetitions of exercise separated by a time interval of 72 h. We aimed to determine whether these subsequent exercise sessions led to the adaptation of the PON1 enzyme response to exercise. Furthermore, correlations between ARE activity and physiological parameters and biochemical variables measured during exercise were searched for.

## 2. Materials and Methods

### 2.1. Subjects

The recruitment process was designed to search for volunteers who met the inclusion criteria, i.e., 25- to 45-year-old men with a training period of 10 to 15 years.

The exclusion criteria were as follows: current alcohol or drug abuse; cigarette smoking habit; infectious or inflammatory disease; systemic treatment with medication, or supplements known to affect the antioxidant status or lipid metabolism.

The study’s sample size was planned based on the calculations of the analysis of variance (ANOVA) test (Statistica Software v13.1, StatSoft Software). For a single comparison of pre- and post-exercise parameters for the sample size of N = 11, the effect size was 1, the power of 0.8, and the alpha value was 0.05. The primary outcome variable for power analysis, which was considered to determine a minimum number of participants to detect significant findings, was the effect of exercise bouts on PON1 concentration.

### 2.2. Study Protocol

**Figure 2:**
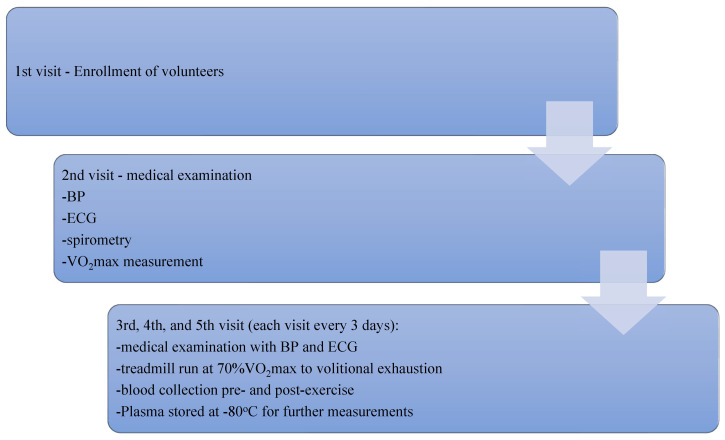
Flowchart of the study. BP—blood pressure measurement, ECG—electrocardiography, VO_2_max—maximal oxygen consumption.

The study was composed of four visits (on observation days 1, 7, 10, and 13) (see Figure 2). During the study period (13 days), subjects were instructed to avoid exhaustive exercise except for the experiment. We specifically asked the participants not to change their usual dietary patterns. The treadmill runs were performed after a light meal. A physical examination, including a resting ECG and blood pressure measurement was performed at each visit. Preliminary testing on day 1 included spirometry tests and a continuous incremental treadmill run on a Trackmaster CP 425 treadmill. The VO_2_max of the subjects was assessed by the gas exchange analysis system Ultima CardiO2 PFX. The exhaustion protocol used to determine maximal aerobic power was a ramp test designed to elicit a test duration of 8–12 min [34]. The treadmill had a static inclination of 1.5%, and its speed was increasing by 1.5 km/h every 3 min, starting at 6 km/h. Throughout the treadmill run, a standard 12-lead wireless ECG was conducted. On days 7, 10, and 13, each subject performed a treadmill run to volitional exhaustion at a speed corresponding to 70% of personal VO_2_max and a static inclination of the treadmill of 1.5%. Each exercise repetition was performed according to the same protocol. The time interval between subsequent exercise bouts was 72 h. Venous blood samples were drawn from the participants in each exercise repetition twice, pre- and directly post-exercise, into lithium heparin and EDTA Vacutainer tubes (Becton, Dickinson and Company, Franklin Lakes, NJ, USA). A blood count was completed, and plasma was separated from the cells by centrifugation at 3000× *g*, 4 °C, 15 min. The plasma was stored at −80 °C for further analysis. Details of the study design have been presented earlier [23,35].

### 2.3. Determinations in Plasma

Analysis of PON1 activities was performed according to the procedure described by Nakanishi et al. [36] using an Ultrospec III spectrophotometer (Pharmacia LKB) with Spectro-Kinetics Software, Pharmacia LKB Biochrom Ltd., Cambridge, UK. Briefly, ARE activity was measured in serum with dilution buffer containing EPPS after initiating the enzyme–substrate reaction by the addition of substrate solution with phenylacetate. The substrate hydrolysis rate was monitored spectrophotometrically at 510 nm at 37 °C. PON activity was previously assessed in a Tris/HCl buffer with paraoxon as a substrate. The paraoxon hydrolysis rate was determined by measuring p-nitrophenol at 405 nm at 37 °C.

The measurement of other biochemical determinations in plasma used for correlation analysis was as described in the previous study [23]. Briefly, PON1 concentration (PON1c) was assessed using an ELISA Kit for paraoxonase 1 (USCN Life Sciences, Inc., Wuhan, China); FRAP was measured spectrophotometrically by a method described by Benzie and Strain [37]. A complete blood count, total cholesterol concentration (TChol), high-density lipoprotein concentration (HDL-C), low-density lipoprotein concentration (LDL-C), triglyceride concentration (TG), plasma creatine kinase activity (CK), aspartate aminotransferase (AST), alanine aminotransferase (ALT), as well as concentrations of C-reactive protein (CRP), lactic acid, glucose, urea, and creatinine, were assessed with the Bekman Culter Analyzer AU680 in the Diagnostic Laboratory of the Central Clinical Hospital of the Medical University of Lodz, Poland.

### 2.4. Genotyping of the PON1 Gene

The PON1 Q192R (rs662) and L55M (rs854560) polymorphisms were analyzed using commercially available TaqMan SNP Genotyping Assays (Applied Biosystems, Foster City, CA, USA). Genomic DNA was isolated from blood leukocytes using a QIAamp DNA Blood Mini Kit (QIAGEN Inc., Valencia, CA, USA) and protocol. The quantification of DNA was performed on a PicoDrop spectrophotometer (Picodrop Limited, Saffron Walden, UK). The genotypes were determined with Sequence Detection System 2.3 Software (Thermo Fisher Scientific, Waltham, MA, USA). The genotyping procedures were previously described [23]. PON1c and PON1 genotypes were analyzed in the Central Scientific Laboratory of the Medical University in Lodz (CoreLab, Kewdale, WA, Australia).

### 2.5. Chemicals

Triton X-100 was obtained from Serva Feinbiochemica (Heidelberg, Germany), Trizma base was from Fluka (Buchs, Switzerland), and all other reagents were from Sigma Aldrich Chemical (St Louis, MO, USA).

### 2.6. Statistical Analysis

Results were expressed as mean ±SD. Data distribution was tested with the Shapiro–Wilk W test (Statistica Software v13.1, StatSoft Software). Analysis of variance (ANOVA) for repeated observations followed by Scheffe’s post hoc parametric test and Friedman ANOVA followed by a post hoc non-parametric Wilcoxon matched pairs test were applied for the analysis of the data for the three repetitions of exercise. Pair-wise correlations for the analysis of associations between ARE activity and selected parameters of antioxidant defense and metabolic response to exercise were carried out by Spearman’s or Pearson’s two-tailed bivariate analyses depending on the data distribution. Due to a relatively low number of participants (*n* = 11), the direct analysis of correlations between variables at each exercise session was inconclusive. Therefore, the pre-exercise individual data from three exercise bouts were pooled together (*n* = 33) and compared with the post-exercise individual data also pooled together from three exercise bouts (*n* = 33) Statistical significance was set at *p* < 0.05.

## 3. Results

Eleven average-trained men were engaged in the study. The characteristics of the participants are presented in Table 1. The basic physiological parameters monitored during subsequent repetitions of exercise are presented in Table 2, and described elsewhere, as this study is a continuation of the protocol [23,35]. The level of training was constant throughout the study (three 1 h aerobic training sessions and a 1.5 h soccer match per week).

Although each of the three exercise repetitions followed the same design, the subjects adjusted to the form of exercise in the second and third treadmill runs, which resulted in a longer running time and distance compared to their first attempt [23].

Genotyping of the PON1 gene showed that our group of volunteers consisted of three subjects with QQ, seven subjects with QR, and one subject with RR genotype in the Q192R polymorphism. Five subjects with LL, six subjects with LM, and no subjects with the MM genotype in the L55M polymorphism were detected (as shown previously [23]). Due to the uneven allele distribution and a low number of subjects within each genotype, no further statistical analysis regarding the PON1 polymorphism was performed.

### 3.1. The Effect of Three Repeated Bouts of Exercise on ARE Activity and Other Biochemical Variables in Plasma

**Figure 3:**
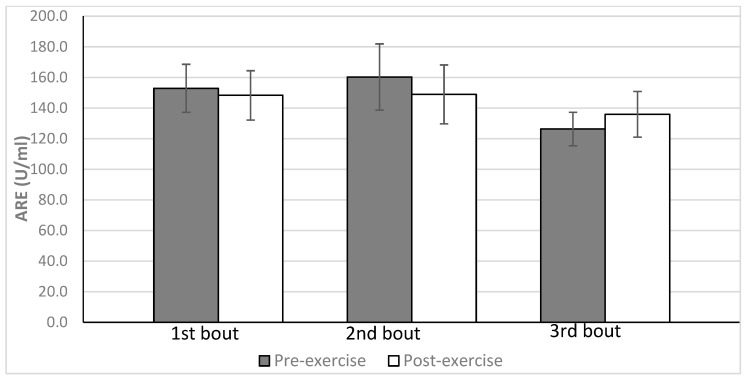
No significant effect of repeated exercise on arylesterase activity (ARE) of paraoxonase 1 (PON1) in the plasma of average-trained men (mean ± SD).

All subjects completed the protocol of three repetitions of strenuous exercise separated by 72 h intervals. No significant differences in ARE activity were observed in any of the three bouts of exercise (Table 3, Figure 3). To verify the observation that exercise did not influence ARE activity, we summed up all pre-exercise measurements and all post-exercise measurements. The statistical analysis of these values confirmed the lack of influence of exercise on ARE activity.

The pre- and post-exercise absolute levels of ARE activity did not change when subsequent visits were compared.

**Figure 4:**
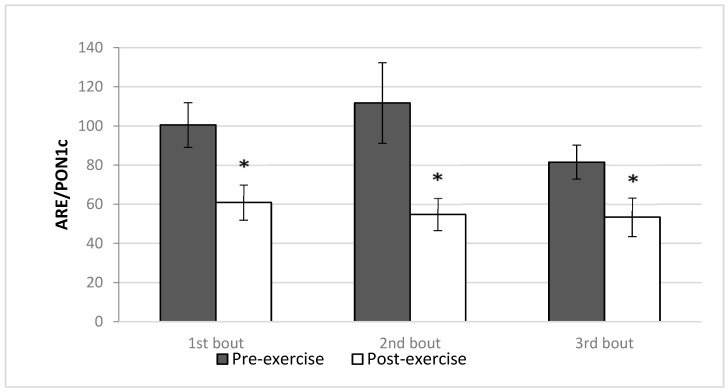
Effect of repeated exercise on arylesterase to paraoxonase 1 concentration ratio (ARE/PON1c) in the plasma of average-trained men (mean ± SD). * vs. corresponding pre-exercise value, *p* < 0.05.

ARE activity standardized for PON1c (ARE/PON1c ratio) was higher pre- than post-exercise in each of the bouts (first and second bout, *p* = 0.002, third bout *p* = 0.03) (Table 3, Figure 4). Comparison of pre- or post-exercise values of ARE/PON1c in subsequent repetitions of exercise did not show differences.

The effect of three repeated bouts of exercise on PON1c, PON activity, HDL-C, TChol, LDL-C, TG, FRAP, and other biochemical variables in plasma was described elsewhere [3]. The most relevant biochemical variables are recalled in Table 3 (for individual data, see Supplementary Tables S1–S3 online).

### 3.2. Correlations between ARE Activity and Other Parameters

**Figure 5:**
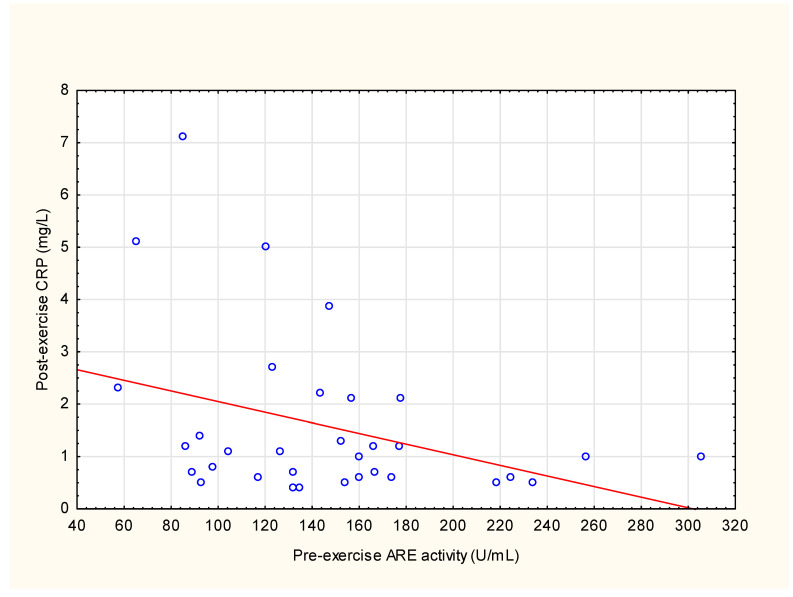
Scatterplot of variables x and y. Correlation of pre-exercise values of arylesterase (ARE) activity and post-exercise C-reactive protein (CRP) in the plasma of average-trained men. Pooled individual data from three exercise bouts (*n* = 33; *ρ* = −0.35, *p* = 0.049).

The correlations of ARE activity with aerobic endurance indices revealed a negative association of post-exercise ARE activity with running distance (*ρ* = −0.37, *p* = 0.042) and time of exercise (*ρ* = −0.37, *p* = 0.042) (see Supplementary Figures S1 and S2). Comparisons of ARE activity with the blood biochemical variables showed a moderate negative correlation between pre-exercise ARE activity and post-exercise CRP (*ρ* = −0.35, *p* = 0.049) (see Figure 5), pre-exercise WBC (*ρ* = −0.38, *p* = 0.03) (see Supplementary Figure S3) and post-exercise WBC (*ρ* = −0.35, *p* = 0.048), post-exercise polymorphonuclear leukocytes (PMN) (*ρ* = −0.37, *p* = 0.037), and post-exercise CK (*ρ* = −0.37, *p* = 0.036) presented in Supplementary Figures S4–S6.

No correlations of ARE activity with VO_2_max, PON1c, PON activity, HDL-C, FRAP, and lactate at exercise were detected.

## 4. Discussion

### 4.1. The Effect of Three Repeated Bouts of Exercise on ARE Activity and Other Biochemical Variables in the Plasma

Regular physical activity is known to offer protection to the cardiovascular system. Subjects who exercise regularly are reported to have a strong antioxidant defense system, as increased amounts of free radicals produced during repeated bouts of exercise upregulate the expression of antioxidant enzymes [38]. However, the mechanisms that lead to the stimulation of endogenous antioxidants resulting from regular repetitions of exercise are still not well understood. In a recent study, we found that a strenuous treadmill run caused an increase in PON1c and PON activity at each repetition of the exercise, with a more pronounced effect of exercise on PON1c [23]. The response of PON1 activity to exercise did not show tolerance. After each exercise session, PON1c and PON activity returned to pre-exercise values. In the current study, which is a continuation of the aforementioned research, we aimed to observe the effect of three repeated bouts of exercise separated by a time interval of 72 h on the ARE activity of the PON1 enzyme. We have previously found that HDL-C increases in exercise bouts [23]. Most of the plasma PON1 is located on the HDL surface. Therefore, we expected that PON1 activity, measured as ARE activity, would also rise as a consequence of the HDL-C increases during exercise. Contrary to our expectations, we observed that acute exercise did not result in changes in ARE activity. These findings support the idea that acute exercise modifies HDL particles, not only by changing their plasma concentration. It may also affect the quality of HDL particles, their composition, and the apolipoproteins attached to them, such as apolipoprotein A-I (Apo A-I). Apo A-I is known to stabilize PON1 [39]. In some conditions, when the Apo A-I concentration in the blood is low, PON1 is transferred from HDL to plasma. PON1 is considered to have lower antiatherogenic activity when it is found in the free fraction of plasma than when it is bound to the HDL particle [40]. In a similar mechanism, factors that influence the level of apolipoprotein E (Apo E) may also influence the activity of PON1 [41].

However, the reaction of PON1 is even more complex. Despite the lack of changes in ARE activity, we have found an increase in PON1c and PON activity at each exercise bout (data previously shown [23]). This dissociation of the effects of acute exercise on PON and ARE activity is not completely surprising. ARE activity has been found to be more stable and less sensitive to different modulating factors than PON activity [42,43]. ARE activity was shown not to be affected by genetic polymorphisms [44]. Arslan et al. found no differences in ARE activity according to training experience, even though there were differences in PON activity levels [45]. It has been described for recombinant PON1 that these two biochemical activities are independent and not closely associated with each other [46]. It is ARE activity, and not PON activity, that correlates best with lactonase activity. Lipolactonase activity is now considered the native activity of PON1, although there is still a lack of clarity on the specificity of the enzyme’s in vivo substrate [46]. Furthermore, ARE activity was shown to be a better marker of the antioxidant activity of PON1 than PON activity [46]. PON1 helps HDL particles prevent the accumulation of lipid peroxides in oxidized LDL. This initial activity may be rapidly consumed and followed by inactivation [47]. The interaction between reactive oxygen species and the free sulfhydryl group in cysteine-284 in the structure of PON1 was found to be responsible for this inactivation after oxidative stress [47,48]. Therefore, we speculate that derangement of oxidative balance during exercise sessions may lead to impairment of ARE activity and its continuous consumption without affecting PON activity as much. PON activity may rise as a consequence of the increase in PON1c, while ARE activity may be continuously used up during exercise sessions. However, even increases in PON activity at the bout of exercise were not as pronounced as PON1c increments, suggesting that PON activity may also be affected by oxidative stress [23]. In addition, we found that longer running time and distance resulted in a lower post-exercise ARE activity. This observation further supports the idea of ARE activity consumption during intense physical activity, especially when exercise is prolonged (see Figure 1). The observed changes in the ARE/PON1c ratio, which was repeatedly higher pre- than post-exercise, support the theory that the absolute value of ARE activity did not fall at exercise bouts due to the increments in PON1c. We speculate that more PON1 molecules were released into the circulation, while the activity of individual particles diminished. However, the effect of ARE activity consumption was not long-lasting, as pre-exercise ARE/PON1c returned to their initial levels in each 72 h interval between exercise sessions. The response of ARE activity to exercise did not show adaptation in subsequent exercise sessions. As there was a difference in the increment of PON1c and the enzyme’s activities, it is important to measure all of these variables in future interventions.

The current observation that ARE activity did not change as a result of any of the exercise sessions is not in agreement with our previous reports, where we had found increments in ARE activity at the bout of exercise [19,21]. However, in those experiments, the sportsmen participated in the maximal exercise, while this current study was conducted at a submaximal exercise of 70% of VO_2_max. The higher load of exercise in the previous setting may also have impacted changes in ARE activity [19,21]. Exercise in our present protocol lasts longer, perhaps leaving more time for the consumption of ARE activity in an environment of oxidative stress. In fact, in our previous experiments, we observed that ARE activity is highest directly during exercise. Measurements taken 2 h after exercise show a return of ARE activity to baseline levels [21]. It is possible that the return of ARE activity to the baseline level after its initial increment occurs sooner, which could have been caught this time, as the exercise lasted longer. A closer observation of the enzyme kinetics during exercise would be beneficial in future studies. We have previously found that changes in ARE activity during exercise depend on the training experience of the subjects. In one study, an increase in ARE activity was observed only in a group of sportsmen who trained for 8–15 years but not in those who trained for 2–7 years [19]. The men in that study were elite rugby players who were very well trained, while this study was conducted in moderately trained men. Furthermore, the men recruited to the present study are older than in our previous experiments (age 34.0 ± 5.2 years in this study in comparison to 17 ± 1 and 21 ± 1 years or 22.0 ± 3.71 years) [19,22], and the age has been shown to affect ARE activity [49].

### 4.2. Correlations between ARE Activity and Other Parameters

Some studies show that intensive bouts of exercise initiate an inflammatory response in the blood. The level of inflammation depends on the type and intensity of exercise, as well as the individual characteristics of the subjects, such as their age and clinical condition [50]. In the present study, we found that ARE activity is inversely correlated with different markers of inflammation. Firstly, a negative correlation was found between pre-exercise ARE activity and post-exercise CRP. CRP is an acute-phase protein released from the liver shortly after the start of a systemic inflammatory process [50]. Increased levels of CRP are a risk factor for cardiovascular disease. CRP is associated with overall morbidity and mortality [50]. CRP increments after intense exercise were described [51,52] and were also recorded in our study after the first exercise session. We speculate that individuals with lower ARE activity at the base level and, therefore, lower protection from oxidative stress by the enzyme, may develop a higher inflammatory response to acute exercise. This matter requires further investigation. Various data describe low PON1 activity in the chronic inflammation state and increased CRP values. In diabetes mellitus, a high plasma CRP is related to low activity of PON1, independently of pro- and anti-inflammatory adipokines [53]. PON1 activity is inversely correlated with CRP in hemodialysis patients [54,55]. Declines in PON1 activity were independently associated with elevated CRP in boys with obesity [56]. Bains et al. demonstrated the effects of chronic and acute inflammation on PON1. They show that PON1 arylesterase and lactonase activity is not only lower in chronic inflammation states, but it also decreases further in the acute inflammatory state [57]. In addition, increased CRP and reduced PON1 have been shown to serve as tools to help detect subjects at higher risk of cardiovascular complications [54,58]. White blood cell count (WBC) is another inflammatory marker that was shown to increase specifically during intense exercise without showing any alteration in moderate exercise settings [59]. The first populations of WBC that are mobilized are PMN [60], while lymphocyte mobilization occurs later, at the end of intense bouts of exercise, and lasts for a short time (about 30 min) [60]. In addition, in our study, we observed an increase in WBC number and PMN after each exercise bout. Furthermore, correlations of ARE activity with blood count revealed an inverse association between pre-exercise ARE and post-exercise WBC and PMN. Finally, we checked the association between ARE and creatine kinase (CK), which is a generally accepted physical stress marker [59]. The CK level increases in cases of muscle fiber damage and signifies the leakage of CK into the plasma. Intense exercise can result in increases in CK [52], which was also observed in our study [23]. It was described that the CK increment after exercise can vary between subjects, but to date, it has not been established what factors modify this response [61]. Interestingly, a negative correlation was observed between pre-exercise ARE activity and post-exercise CK. The above observations suggest that ARE activity may have some protective potential to reduce the development of inflammation and muscle damage caused by intensive exercise.

### 4.3. The Study Limitations

The relatively low number of participants (*n* = 11) is the main weakness of the study. However, when planning this experiment, we focused on showing large effects. Unfortunately, as a consequence of the group size, it was not possible to study the effects of PON1 polymorphisms on ARE activity. It would have added additional information to the study and should be considered in future research. When testing for correlations, in order to use data from all exercise repetitions, we pooled data from three exercise bouts. This led to the observation of interesting correlations between some measurements. Although these results point to some associations, they should be treated with caution, as they could be affected by an error related to the within-participant effect. The study should be repeated with a larger group. As we now observe the direction of changes, more data would bring us closer to final conclusions. Unfortunately, a study of this design with three repetitions of strenuous exercise is very demanding for the participants, and hence difficult to perform on a larger scale.

Another flaw of the study is the lack of female representation in the group. However, we felt that estrogen changes during the menstrual cycle could be a confounding factor in this experiment since estrogen was previously shown to have a stimulating effect on the PON1 enzyme [62].

## 5. Conclusions

We postulate that strenuous exercise may have a detrimental effect on enzyme activity. PON1 concentration increments after subsequent exercise repetitions were not paralleled by increases in ARE activity. The repeated decreases in the ARE/PON1c ratio at each exercise bout with a return to the pre-exercise level at rest suggest that some compensatory mechanisms are employed, which help regain ARE activity after its initial loss during exercise. Perhaps awakening these systems of reparation stimulates endogenous antioxidant defense in the long run if regular physical activity is implemented.

Overall:

1. Dissociation is present between PON1 concentration and ARE activity after strenuous exercise.
2. ARE activity may be depleted and consumed under conditions of oxidative stress related to strenuous exercise.
3. ARE might influence the intensity of the inflammatory response and muscle damage in response to strenuous exercise.

## Figures and Tables

**Table 1:** Characteristics of the participants (mean ± SD).

	Participants Characteristics (N = 11)
Age (years)	34.0 ± 5.2
Gender	male
Race	Caucasian
Training experience (years)	10–15
Body mass (kg)	87.4 ±13.8
BMI (kg/m^2^)	26.2 ± 3.1
VO_2_max (ml/kg/min)	49.6 ± 4.5
Resting HR (beats/min)	72 ± 11
Resting BP (mmHg)	127 ± 6/80 ± 4
FVC (L)	6.09 ± 0.41
% of predicted FEV1	106.4 ± 6.4
FEV1/FVC (%)	80.9 ± 5.6

BMI—body mass index, VO_2_max—maximal oxygen uptake, HR—heart rate, BP—blood pressure, FVC—forced vital capacity, FEV1—forced exhaled volume in the first second.

**Table 2:** Parameters monitored during three bouts of exercise (mean ± SD).

Parameter	Exercise Bout		
	1st Bout	2nd Bout	3rd Bout
HRmax (beats/min)	184 ± 10	183 ± 11	176 ± 12 *^
Post-exercise BP (mmHg)	172 ± 20/82 ± 11	169 ± 13/80 ± 10	166 ± 13/79 ± 11
Running distance (km)	8.6 ± 5.5	10.7 ± 7.6 *	10.4 ± 7.2 *
Running time (min)	47 ± 31	57 ± 41 *	56 ± 40 *

HRmax—maximal heart rate, BP—blood pressure. * vs corresponding value at 1st bout, *p* < 0.05. ^ vs corresponding value at 2nd bout, *p* < 0.05.

**Table 3:** Biochemical measurements in blood (mean ± SD).

Biochemical Measurements	First Exercise		Second Exercise		Third Exercise	
	Before	Bout	Before	Bout	Before	Bout
Arylesterase activity (ARE) (U/mL)	152.9 ± 15.7	148.3 ± 16.1	160.3 ± 21.6	148.9 ± 19.2	126.3 ± 10.9	135.9 ± 15
Paraoxonase activity (PON) (U/L)	884.4 ± 96.2	941.8 ± 93.1 *	831 ± 83.8	914.6± 98.8 *	858.2 ± 83.5	964 ± 86.8 *
Paraoxonase 1 concentration (PON1c) (µg/mL)	1.62 ± 0.14	2.67 ± 0.2 *	1.55 ± 0.11	2.99 ± 0.25 *	1.61 ± 0.1	2.85 ± 0.26 *
Arylesterase activity/paraoxonase 1 concentration (ARE/PON1c)	100.5 ± 37.8	60.9 ± 29.9 *	111.7 ± 68.3	54.8 ± 29.3 *	81.5 ± 28.5	54.8 ± 32.8 *
Ferric-reducing activity of plasma (FRAP) (mM/L Fe^+2^)	1.15 ± 0.06	1.34 ± 0.07 *	1.07 ± 0.05	1.29 ± 0.06 *	1.18 ± 0.05	1.32 ± 0.04 *
High-density lipoprotein (HDL) (mM/L)	1.34 ± 0.05	1.45 ± 0.05 *#	1.29 ± 0.05	1.39 ± 0.06 *	1.32 ± 0.04	1.4 ± 0.05 *
C-reactive protein (CRP) (mg/L)	0.99 ± 0.19	1.53 ± 0.41 *	1.31 ± 0.35	1.29 ± 0.41	1.73 ± 0.56	1.92 ± 0.60
Lactate (mM/L)	1.72 ± 0.24	8.95 ± 1.46 *	1.42 ± 0.21	8.83 ± 1.59 *	1.56 ± 0.15	7.97 ± 1.42 *
Creatine kinase (CK) (U/L)	162.2 ± 20.1	210.8 ± 35.5 *	266.6 ± 68.4	301.8 ± 58.3	300.8 ± 41.7	348.4 ± 41.0 *
White blood cells (WBC) (×10^3^/mm^3^)	5.86 ± 0.62	9.45 ±1.94 *	5.68 ± 0.59	9.99 ± 2.87 *	5.76 ± 0.49	10.7 ± 3.24 *
polymorphonuclear leukocytes (PMN) (×10^3^/mm^3^)	3.98 ± 0.71	6.02 ± 1.88 *	3.85 ± 0.68	6.46 ± 2.92 *	3.97 ± 0.60	7.27 ± 3.49 *

* vs. corresponding pre-exercise value, *p* < 0.05. # vs. corresponding post-exercise value of second or third exercise bout, *p* < 0.05.

## Data Availability

Data is contained within the article and Supplementary Material.

## Supplementary Materials

The following supporting information can be downloaded at: https://www.mdpi.com/article/10.3390/antiox12061296/s1, Supplementary Table S1. Individual data of pre- and post-exercise arylesterase activity (ARE) in average-trained men at each of the three exercise repetitions. Supplementary Table S2. Individual data of pre- and post-exercise paraoxonase 1 concentration (PON1c) in average-trained men at each of the three exercise repetitions., Supplementary Table S3. Individual data of pre- and post-exercise arylesterase/paraoxonase 1 concentration (ARE/PON1c) in average-trained men at each of the three exercise repetitions. Supplementary Table S4. Biochemical measurements data distribution tested using Shapiro–Wilk test with visual inspection of histograms (*n* = 11). Supplementary Table S5. Exercise parameters data distribution tested using Shapiro–Wilk test with visual inspection of histograms (*n* = 11), Supplementary Figure S1. Scatterplot of variables x and y. Correlation of post-exercise values of arylesterase (ARE) activity and running distance in the plasma of average-trained men. Supplementary Figure S2. Scatterplot of variables x and y. Correlation of post-exercise values of arylesterase (ARE) activity and running time in the plasma of average-trained men. Supplementary Figure S3. Scatterplot of variables x and y. Correlation of pre-exercise values of arylesterase (ARE) activity and pre-exercise white blood cells (WBC) in the plasma of average-trained men. Supplementary Figure S4. Scatterplot of variables x and y. Correlation of pre-exercise values of arylesterase (ARE) activity and post-exercise white blood cells (WBC) in the plasma of average-trained men. Supplementary Figure S5. Scatterplot of variables x and y. Correlation of pre-exercise values of arylesterase (ARE) activity and post-exercise polymorphonuclear leukocytes (PMN) in the plasma of average-trained men. Supplementary Figure S6. Scatterplot of variables x and y. Correlation of pre-exercise values of ARE activity and post-exercise creatine kinase (CK) in the plasma of average-trained men.
