# Supplementary material for: Effect of Exercise Repetitions on Arylesterase Activity of PON1 in Plasma of Average-Trained Men—The Dissociation between Activity and Concentration
Source: Antioxidants (Basel). 2023 Jun 17;12(6):1296. doi: 10.3390/antiox12061296 (PMC10295568; doi:10.3390/antiox12061296)
Supplement: Supplementary file 1 [file antioxidants-12-01296-s001.zip › antioxidants-2410041-supplementary.pdf]

Supplementary material for

Effect of exercise repetitions on arylesterase activity of PON1 in plasma of average-trained men – dissociation between activity and concentration

Aneta Otocka-Kmiecik, Monika Orłowska-Majdak, Robert Stawski, Urszula Szkudlarek, Gianluca Padula, Szymon Gałczyński, Dariusz Nowak

Supplementary Table S1. Individual data of pre- and post-exercise arylesterase activity (ARE) in average-trained men at each of the three exercise repetitions

	Participant number	1st bout		2nd bout		3rd bout	
		Pre-exercise	Post-exercise	Pre-exercise	Post-exercise	Pre-exercise	Post-exercise
**ARE (U/mL)**	1	92.7	63.7	89.1	90.7	57.5	91.2
	2	153.9	130.6	234.2	242.5	160.1	160.6
	3	123.3	187.0	177.7	188.1	143.5	171.0
	4	166.3	128.5	152.3	134.7	126.4	81.9
	5	224.9	165.8	218.7	239.4	174.1	178.2
	6	177.2	229.5	305.7	190.7	104.7	232.1
	7	117.1	211.9	134.7	161.1	132.1	148.2
	8	120.7	164.2	65.3	159.6	147.7	135.2
	9	256.5	167.9	166.8	97.4	160.1	139.9
	10	157.0	114.0	132.1	82.9	85.0	79.3
	11	92.2	67.9	86.5	50.8	97.9	77.2

Supplementary Table S2. Individual data of pre- and post-exercise paraoxonase 1 concentration (PON1c) in average-trained men at each of the three exercise repetitions

	Participant number	1st bout		2nd bout		3rd bout	
		Pre-exercise	Post-exercise	Pre-exercise	Post-exercise	Pre-exercise	Post-exercise
**PON1C (µg/mL)**	1	1,55	2,95	1,28	1,99	1,32	2,21
	2	1,19	2,15	1,14	2,22	1,24	2,2
	3	1,42	2,86	1,3	3,42	1,39	3,59
	4	1,46	2,7	1,56	3,67	1,64	2,91
	5	1,62	2,66	2,04	3,33	1,93	3,92
	6	1,21	2,03	1,15	1,99	1,31	1,66
	7	1,51	1,96	1,62	2,43	1,68	2,58
	8	1,29	3,15	1,28	3,4	1,36	3,97
	9	1,74	2,42	1,57	2,46	1,65	2,13
	10	1,98	2,12	2,24	3,31	2,21	2,23
	11	2,83	4,31	1,84	4,63	1,95	3,96

Supplementary Table S3. Individual data of pre- and post-exercise arylesterase / paraoxonase 1 concentration (ARE/PON1c) in average-trained men at each of the three exercise repetitions

	Participant number	1st bout		2nd bout		3rd bout	
		Pre-exercise	Post-exercise	Pre-exercise	Post-exercise	Pre-exercise	Post-exercise
ARE/PON1c	1	59.8	21.6	69.6	45.6	43.6	41.3
	2	129.3	60.7	205.4	109.2	129.1	73.0
	3	86.8	65.4	136.7	55.0	103.3	47.6
	4	113.9	47.6	97.7	36.7	77.1	28.1
	5	138.8	62.3	107.2	71.9	90.2	45.5
	6	146.4	113.1	265.8	96.0	79.9	139.8
	7	77.5	108.1	83.2	66.3	78.7	57.4
	8	93.6	52.1	51.0	46.9	108.6	34.1
	9	147.4	69.4	106.3	39.6	97.0	65.7
	10	79.3	53.8	59.0	25.0	38.5	35.6
	11	32.6	15.8	47.0	11.0	50.2	19.5

Supplementary Table S4. Biochemical measurements data distribution tested using Shapiro-Wilk test with visual inspection of histograms (n=11)

Biochemical measurements	1st bout		2nd bout		3rd bout	
	Pre-exercise	Post-exercise	Pre-exercise	Post-exercise	Pre-exercise	Post-exercise
	W (p)	W (p)	W (p)	W (p)	W (p)	W (p)
Arylesterase activity (ARE) (U/mL)	0.92, (0.32), N	0.95, (0.70), N	0.96, (0.72), N	0.95, (0.64), N	0.95, (0.68), N	0.92, (0.32), N
Paraoxonase activity (PON) (U/L)	0.84, (0.04), NN	0.80, (0.01), NN	0.92, (0.29), N	0.93, (0.45), N	0.80,(0.01), NN	0.93, (0.37), N
Paraoxonase 1 concentration (PON1c) (μg/mL)	0.80, (0.01), NN	0.87, (0.08), N	0.91, (0.25), N	0.91, (0.28), N	0.91, (0.24), N	0.86, (0.07), N
Aryleserase activity/paraoxonase 1 concentration (ARE/PON1c)	0.94, (0.54), N	0.92, (0.31), N	0.84, (0.03), NN	0.96, (0.81), N	0.96,(0.73), N	0.81, (0.01), NN
Ferric reducing activity of plasma (FRAP) (mM/L Fe^{+2})	0.95, (0.58), N	0.89, (0.13), N	0.95, (0.61), N	0.98, (0.94), N	0.95, (0.61), N	0.99, (0.99), N
High-density lipoprotein (HDL) (mM/L)	0.91, (0.27), N	0.95, (0.65), N	0.75, (0.00), NN	0.81, (0.01), NN	0.85,(0.04), NN	0.89, (0.15), N
C-reactive protein (CRP) (mg/L)	0.84, (0.03), NN	0.76, (0.003), NN	0.70, (0.01), NN	0.64, (0.00), NN	0.7, (0.01), NN	0.74, (0.00), NN
Lactate (mM/L)	0.92, (0.35), N	0.92, (0.35), N	0.84, (0.04), NN	0.93, (0.43), N	0.87, (0.08), N	0.94, (0.47), N
Creatine kinase (CK) (U/L)	0.84, (0.03), NN	0.67, (0.000), NN	0.81, (0.01), NN	0.84, (0.03), NN	0.97, (0.90), N	0.91, (0.24), N
White blood cells (WBC) ($x10^3/mm^3$)	0.75, 0.002, NN	0.94, (0.47), N	0.94, (0.50), N	0.82, (0.02), NN	0.92, (0.29), N	0.87, (0.08), N
Polymorphonuclear leukocytes (PMN) ($x103/mm^3$)	0.92, 0.34, N	0.88, (0.10), N	0.95, (0.58), N	0.86, (0.05), NN	0.93, (0.40), N	0.87, (0.07), N

W – the value of the test statistics, (p) – p-value, N- normal distribution, NN-not normal distribution

Supplementary Table S5. Exercise parameters data distribution tested using Shapiro-Wilk test with visual inspection of histograms (n=11)

**Parameter**	**Exercise bout**		
	**1st bout**	**2nd bout**	**3rd bout**
	**W (p)**	**W (p)**	**W (p)**
**Maximal heart rate (HR) during exercise (beats/min)**	0.92, (0.29), N	0.85, (0.048), NN	0.90, (0.18), N
**Post-exercise systolic blood pressure (SBP) (mmHg)**	0.89, (0.14), N	0.90, (0.21), N	0.62, (0.0001), NN
**Post-exercise diastolic blood pressure (DBP (mmHg)**	0.91, (0.29), N	0.81, (0.01), NN	0.83, (0.02), NN
**Running distance (km)**	0.88, (0.11), N	0.86, (0.051), N	0.80, (0.01), NN
**Running time (min)**	0.89, (0.13), N	0.84, (0.03), NN	0.80, (0.01), NN

W – the value of the test statistics, (p) – p-value, N – normal distribution, NN – not normal distribution

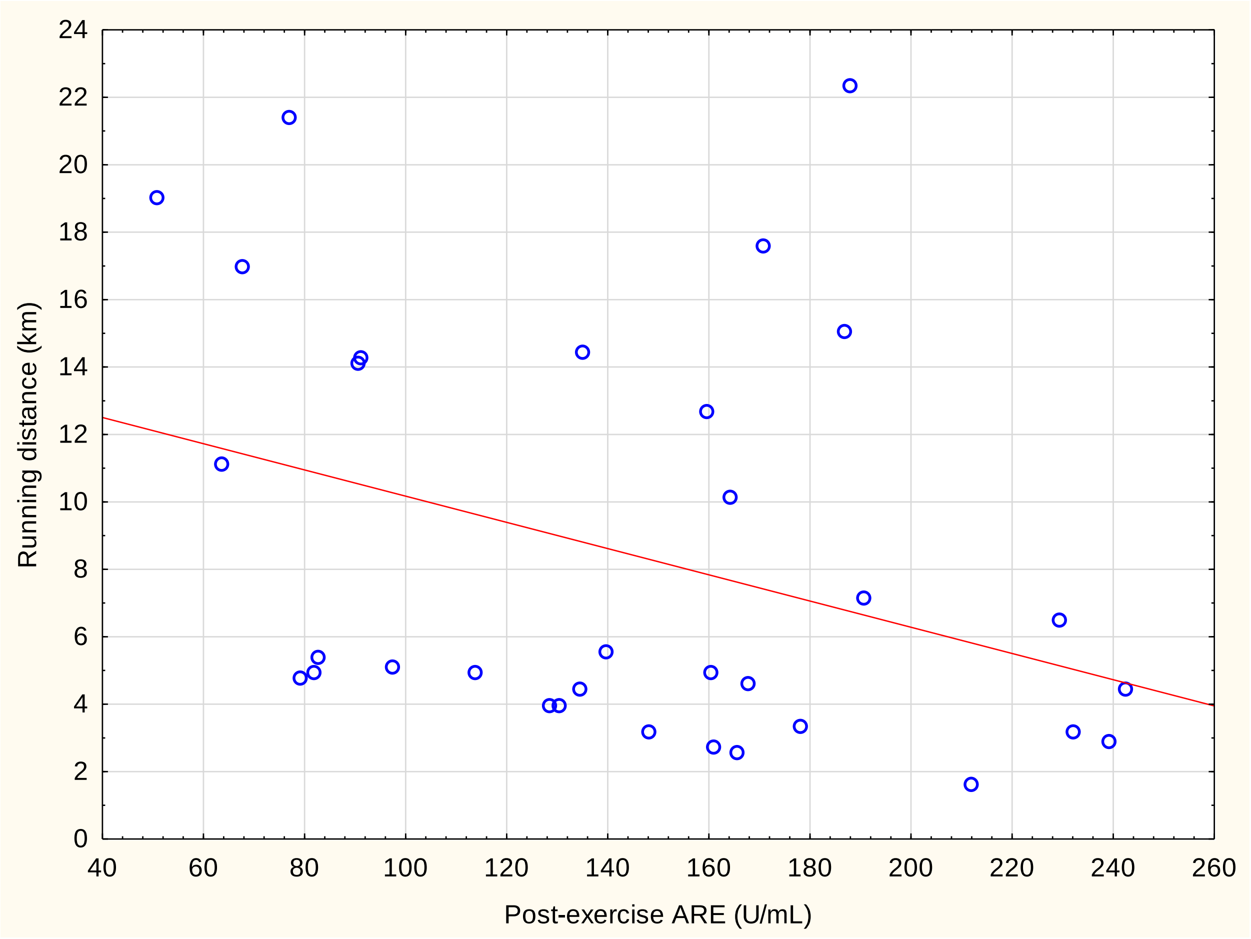

Supplementary Figure S1. Scatterplot of variables x and y. Correlation of post-exercise values of arylesterase (ARE) activity and running distance in the plasma of average trained men. Pooled individual data from three exercise bouts (n=33; ρ=-0.37, p=0.042)

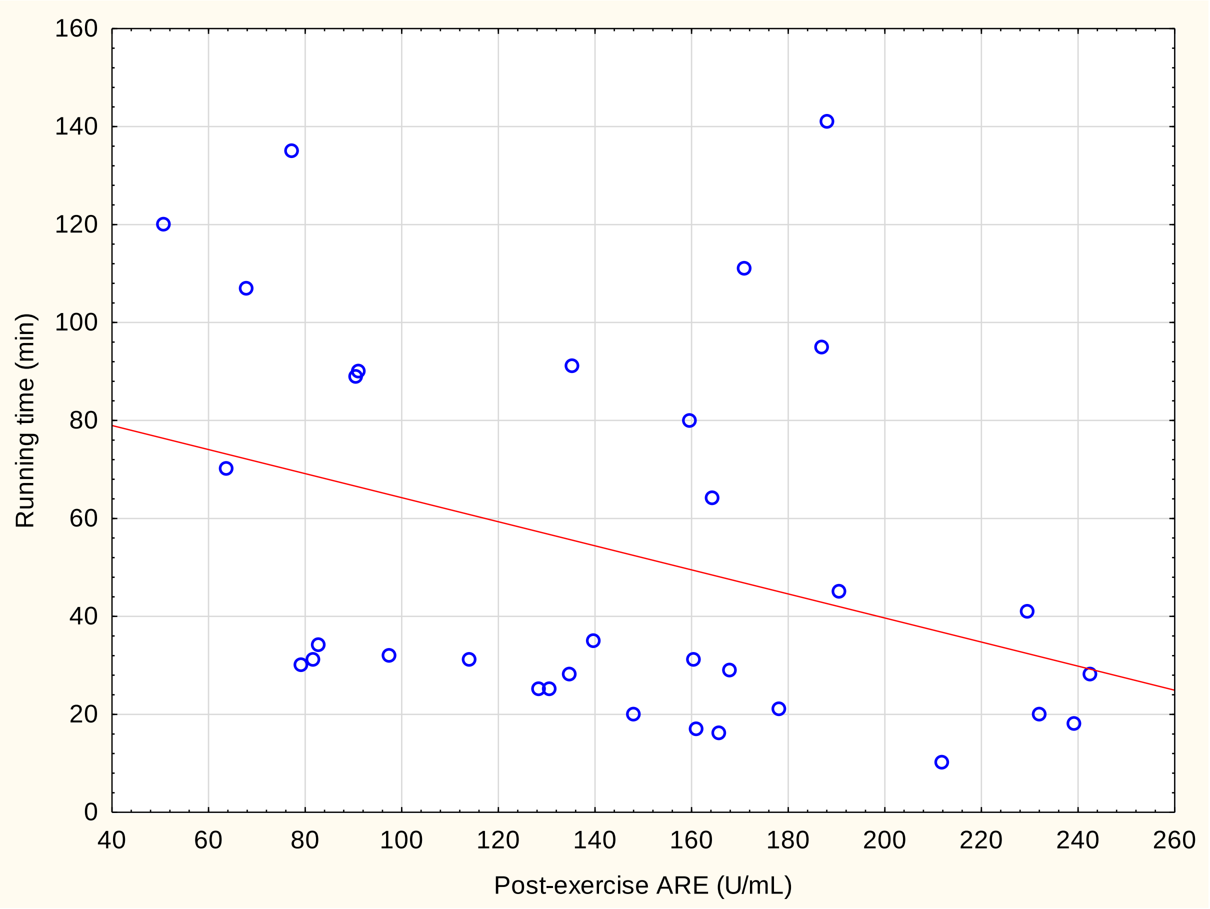

Supplementary Figure S2. Scatterplot of variables x and y. Correlation of post-exercise values of arylesterase (ARE) activity and running time in the plasma of average trained men. Pooled individual data from three exercise bouts (n=33; ρ=-0.37, p=0.042)

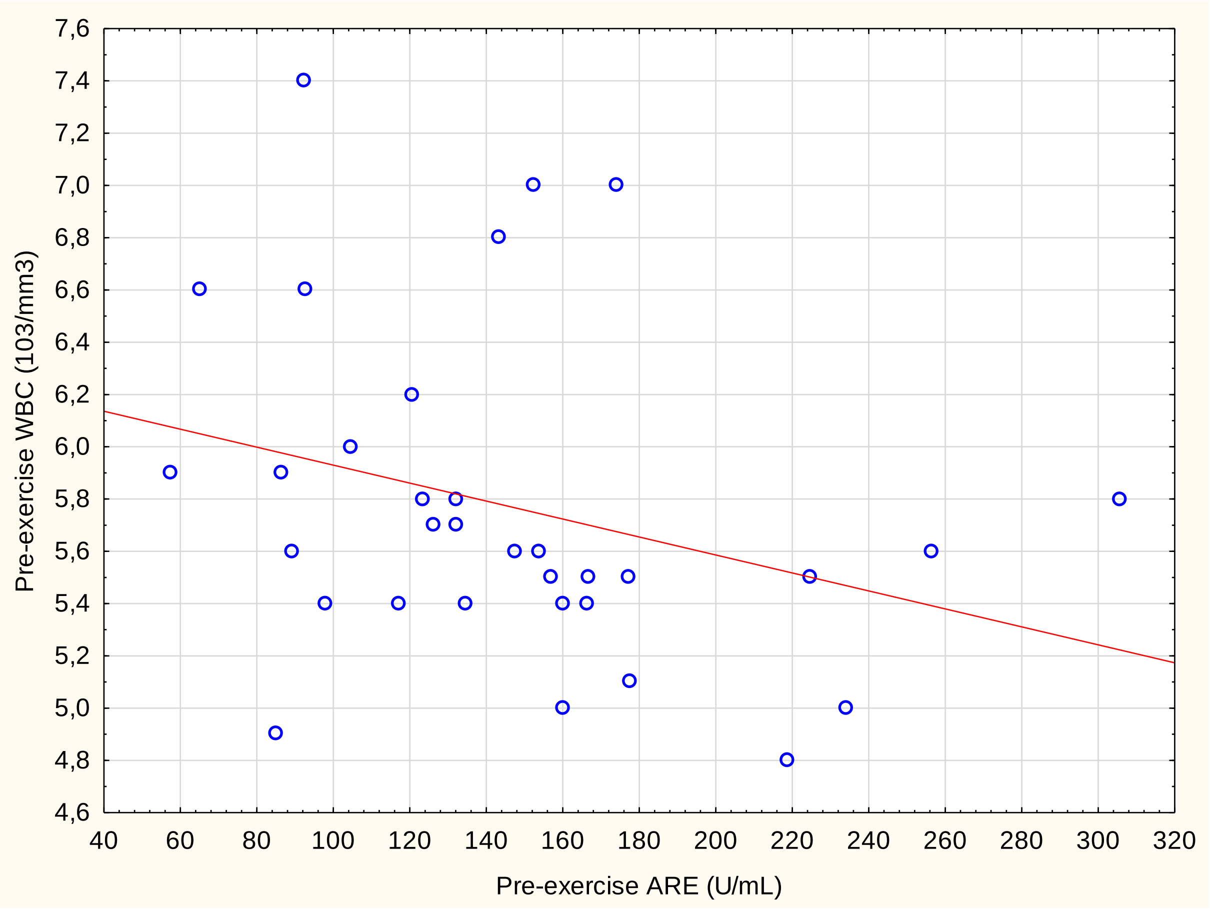

Supplementary Figure S3. Scatterplot of variables x and y. Correlation of pre-exercise values of arylesterase (ARE) activity and pre-exercise white blood cells (WBC) in the plasma of average trained men. Pooled individual data from three exercise bouts (n=33; ρ=-0.38, p=0.03)

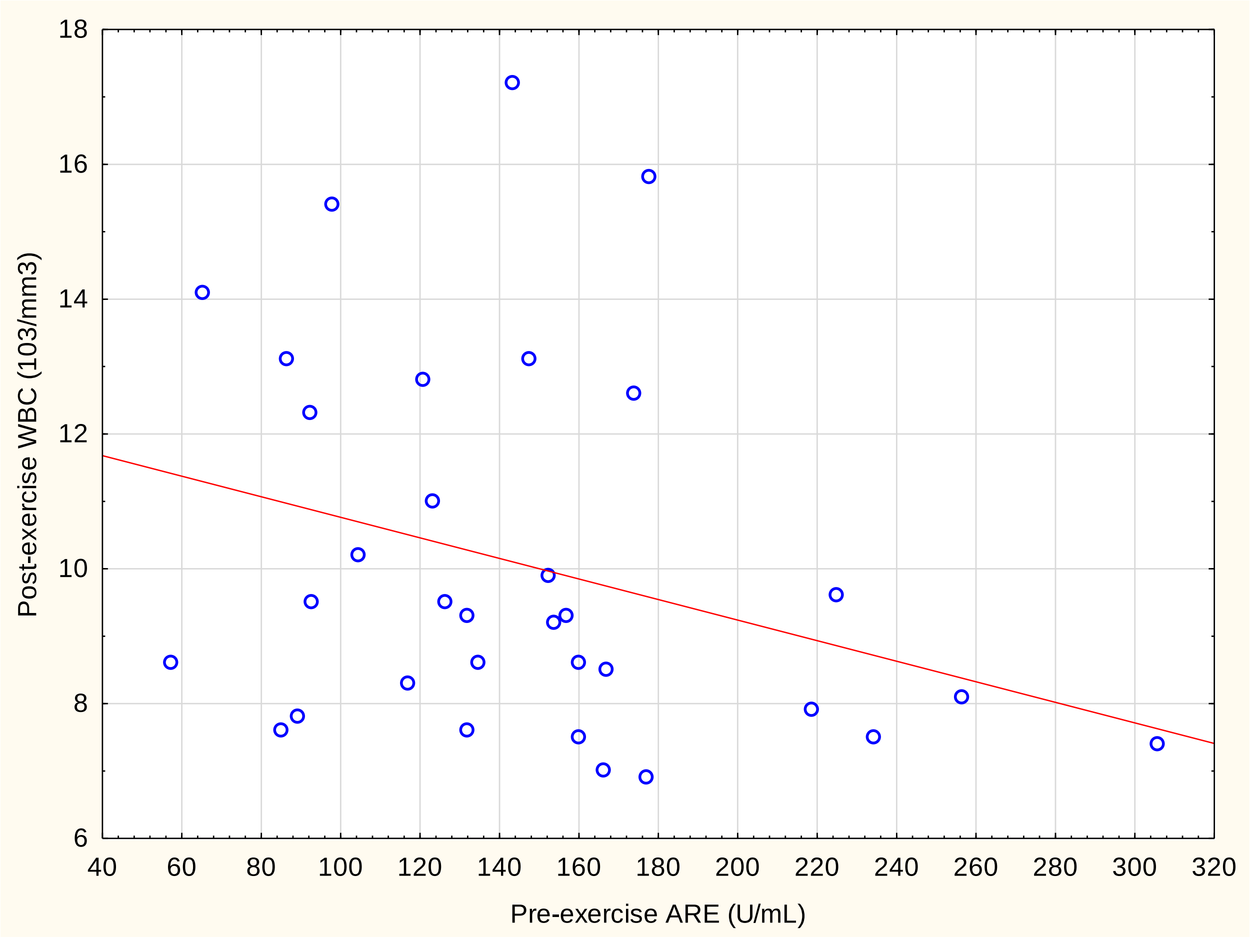

Supplementary Figure S4. Scatterplot of variables x and y. Correlation of pre-exercise values of arylesterase (ARE) activity and post-exercise white blood cells (WBC) in the plasma of average trained men. Pooled individual data from three exercise bouts (n=33; ρ=-0.35, p=0.048)

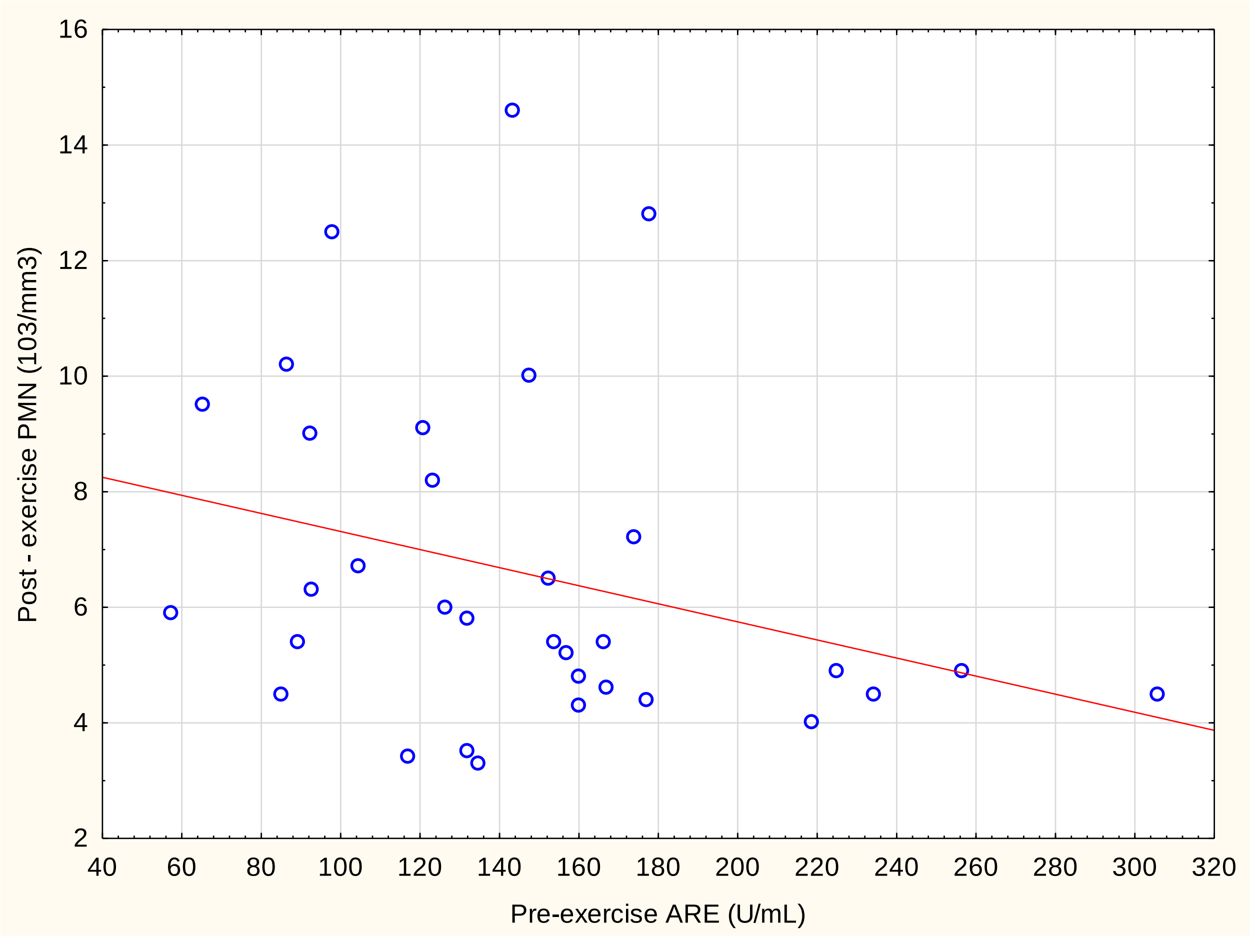

Supplementary Figure S5. Scatterplot of variables x and y. Correlation of pre-exercise values of arylesterase (ARE) activity and post-exercise polymorphonuclear leukocytes (PMN) in the plasma of average trained men. Pooled individual data from three exercise bouts (n=33; ρ=-0.37, p=0.037)

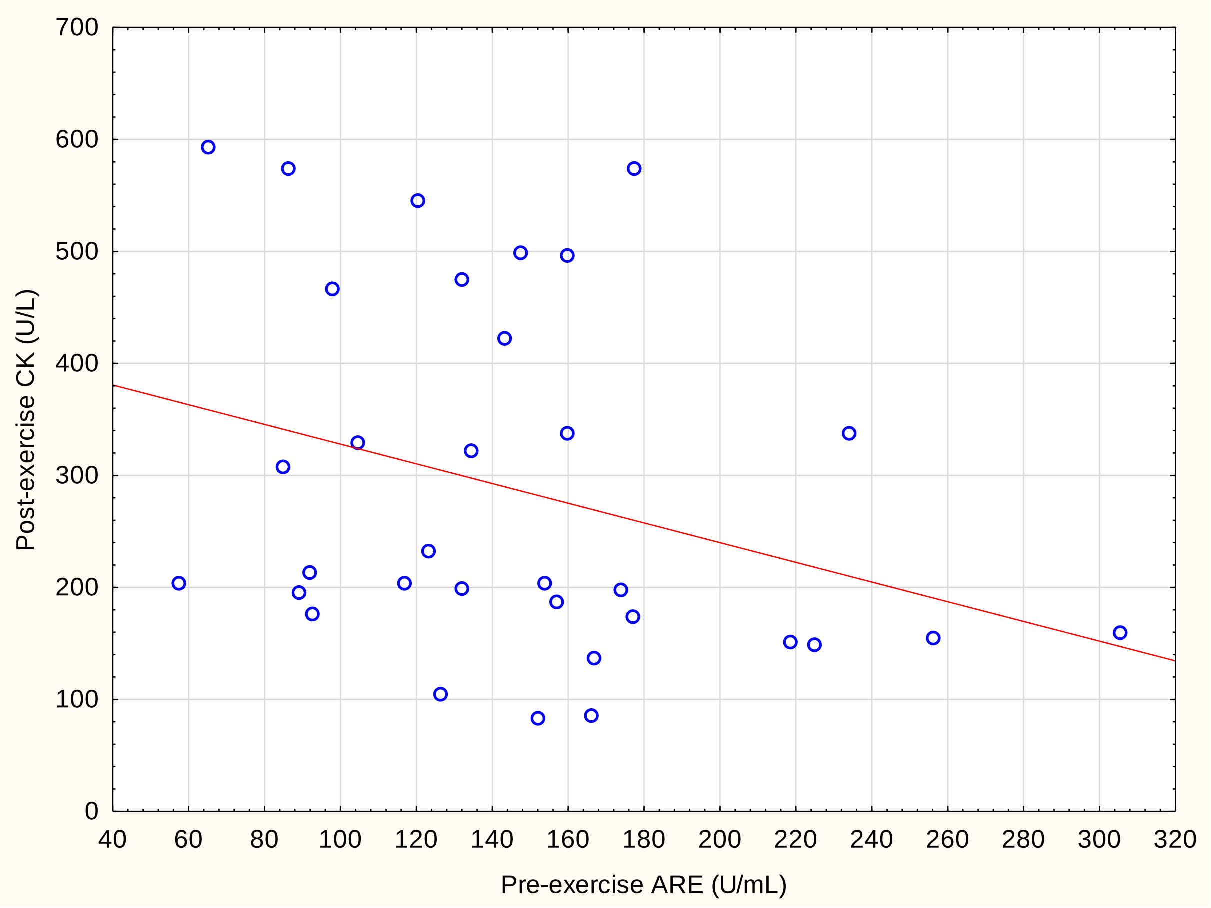

Supplementary Figure S6. Scatterplot of variables x and y. Correlation of pre-exercise values of ARE activity and post-exercise creatine kinase (CK) in the plasma of average trained men. Pooled individual data from three exercise bouts (n=33; ρ=-0.37, p=0.036)
